# Investigations Into the Impact of Static Magnetic Fields on Blood Flow

**DOI:** 10.7759/cureus.78007

**Published:** 2025-01-26

**Authors:** Harvey N Mayrovitz

**Affiliations:** 1 Medical Education, Nova Southeastern University Dr. Kiran C. Patel College of Allopathic Medicine, Davie, USA

**Keywords:** blood circulation, blood flow, blood velocity, magnetic field, magnetic therapy, magnets, permanent magnet, review article

## Abstract

Many claims are made regarding the impacts of static magnetic fields (SMFs) on biological and physiological processes. Some of these are based on scientific underpinnings, and others appear to have less evidence to support them. The present report focuses on the evidence regarding SMF's effects on blood flow. Fortuitously, the author has direct experimental experience in this area. The approach for this review was to search three major databases (Web of Science, PubMed, and Embase) for peer-reviewed articles written in English in which an SMF was used in humans or other animals and measurements of parameters related to blood flow or velocity before SMF application and either during or after application were reported. After screening the initial 1,954 articles, 108 studies were retrieved and evaluated for relevancy. Of these, 23 were found to satisfy the inclusion criteria and be relevant. This included 10 studies on humans and 13 studies on other animals. The methods employed in many of these studies are illustrated in this review to enhance understanding of the findings. With regard to human studies, none showed an increase in blood flow, and one showed a decrease in flow. With regard to the animal studies, one showed a transient post-exposure increase that was later explained as due to an actual reduction during SMF exposure. Four studies showed a decrease, four showed no change or difference from sham-exposed animals, and four reported an increase. Of these four, two were from the same author using a method that may not have reflected a blood flow change. Based on these findings, it is concluded that claims of an SMF providing an increase in blood flow or circulation are not supported by human studies and not well supported by animal studies. However, this does not close the door to a possible effect for at least four considerations or limitations that may have impacted the absence of a positive finding in human studies: (1) the number of subjects included is relatively small, which affects the study power; (2) the duration of the SMF application of most studies was relatively short; (3) most studies were done on healthy individuals; and (4) the SMF was delivered perpendicular to the body surface, so the effects of tangential field directions are unknown. Although these provisos may impact the detection of a possible SMF effect, they do not alter the current findings, as no reviewed human study has demonstrated a statistically significant increase in blood circulation attributable to an SMF. Thus, the clinical use of an SMF to improve blood circulation is not supported by experimental evidence.

## Introduction and background

A static magnetic field (SMF) is produced by a steady, non-time-varying electrical current or a permanent magnet. Both forms have been used to investigate the possible impacts of SMFs on various biological processes and clinical conditions. The breadth of targets studied has been extensive. The potential role of SMF in treating pain of various origins has received much attention but with varying results [1-5]. Other studies have focused on an SMF's potential utility in diabetes [6-8], wound healing [9-11], and a host of other conditions [12-15]. There are also reports concerning using SMF to affect brain functions via transcranial applications [16-18]. Some scientific reports have ruled out SMF efficacy concerning some aspects of pain [19,20], and others have severely criticized SMF as therapy [21,22]. This type of criticism is in part directed toward claims that are non-scientifically supported and particularly observable in some commercial advertisements and some non-peer-reviewed softcover books that offer lists of conditions that could be helped by SMF therapy [23-30]. Although some of the text in these volumes provides pertinent references, much is speculative. One claim, which is the main focus of the present study, is that an SMF can promote increased blood circulation or blood flow. Examples claiming such benefits can be found in the text of books [23,25,29,30] and on multiple websites. For such a claim to be accepted, sufficient direct evidence of a blood flow, blood velocity, or blood perfusion increase in humans or experimental animals exposed to an SMF would be needed. Some scientific studies have assessed the impact of SMFs on vascular tone and vasomotion in experimental animals [31-35]. However, any link between such vascular changes and increased blood flow would be speculative. The significance of increased blood flow in many aspects of therapy makes it an important topic of study. Thus, the present study focuses only on those studies in which actual blood flow, velocity, or perfusion parameters have been measured before and either during or after the application of an SMF. The main aim is to present and discuss the available evidence of a linkage between the application of an SMF and a blood flow increase.

## Review

Methods

Databases Searched

Three databases (Web of Science, PubMed, and Embase) were searched for peer-reviewed published articles in English through 12/2/2024.

Search Method

**Figure 1:**
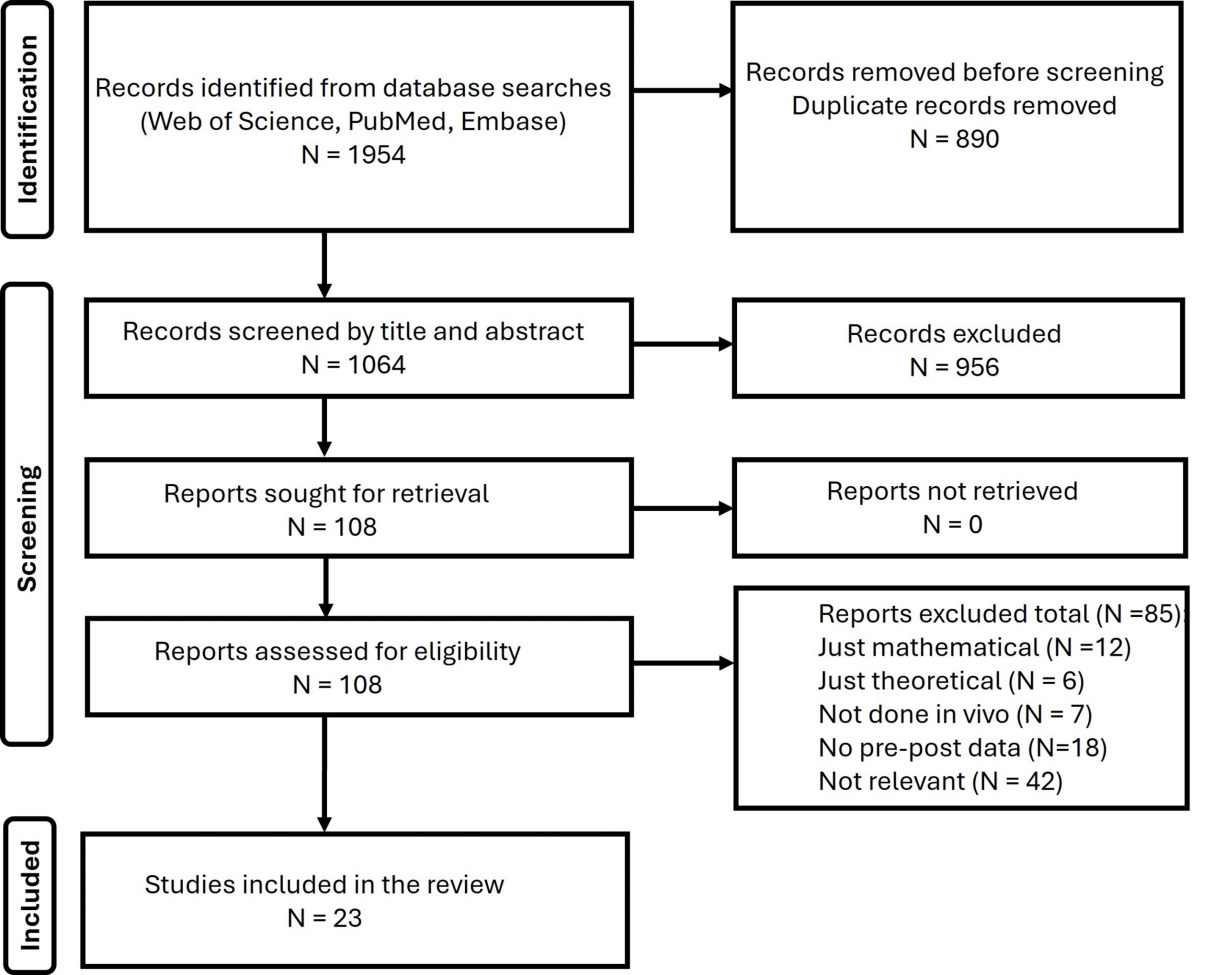
Search sequence

For the Web of Science search, the initial Boolean search string was as follows: (static magnet* OR permanent magnet* OR steady magnet* OR constant magnet*) AND (blood flow OR blood perfusion OR blood velocity OR laser Doppler). This yielded 3,051 citations. A follow-up modified search string incorporated terms to exclude irrelevant citations if they were related to pumping, imaging, or MRI use. The final search term was (static magnet* OR permanent magnet* OR steady magnet* OR constant magnet*) AND (blood flow OR blood perfusion OR blood velocity OR laser Doppler) NOT (pump OR imag* OR MRI). This reduced the total number of citations found on the Web of Science to 987. These were then filtered to accept only full articles for a final count of 904. A corresponding search string was used in the PubMed database to yield 455 articles and in Embase 595 articles for a total of 1,954 records, as shown in Figure 1.

These 1,954 records were examined for duplicates, and 890 were found and removed, leaving 1,064 records. These were reviewed by title and, if needed and available, by abstract to assess relevancy. Of the 1,064 records screened, 956 were excluded as irrelevant, leaving 108 as potentially relevant. Full versions of these 108 studies were obtained and reviewed. After reviewing the full documents, 85 were eliminated because they were theoretical, just mathematical, were not done in vivo (human or animal), contained no new experimental data, or did not provide data documenting before SMF treatment values and either during or after SMF treatment. Also, 42 were eliminated as irrelevant upon full reading of the report. This left 23 studies to be included in this review.

Studies on humans

Table 1 summarizes the major elements of the 10 studies in which SMF was applied to humans, and some aspects related to blood flow were measured. These studies will be considered in further detail in the discussion section. However, it is notable that the range of measurement methods was variable. Six of the 10 studies estimated skin blood flow (SBF) using the method of laser Doppler flowmetry. This method does not measure blood flow but measures a quantity proportional to blood flow. This quantity is often termed perfusion and is the product of the red blood cell (RBC) volume concentration multiplied by the RBC velocity and by a constant [36-40]. One study uses temperature changes, which indirectly reflect blood flow. The other three studies use methods also recognized as measuring blood flow changes. None of these studies reported an increase, and one indicated a decrease in blood flow.

**Table 1:** Summary of human study results Column 1 shows the first author, year of publication, and reference number. SMF is the static magnetic field applied.

First author, year, Ref #	Subjects studied (N)	SMF source	SMF surface intensity	Application site	Application duration	Flow measurement method	Flow measurement site	Mean blood flow change	Comment
Barker, 1985 [41]	10	Magnetized foil	40 mT	Medial forearm	7 hours	Temperature change	Medial forearm	None	Sham arm vs. active magnet on the other arm
Saygili, 1992 [42]	10	Cobalt-samarium magnets	95 mT	Buccal mucosal	45 days	^133^Xe clearance	Buccal mucosal	None	Placebo vs. active magnet
Mayrovitz, 2001 [43]	16	Ceramic magnets	100 mT	Hand thenar eminence	36 minutes	Laser Doppler	Finger dorsum skin	None	Sham vs. active magnet
Martel, 2002 [44]	20	Magnets	50 mT	Anterior forearm	30 minutes	Strain gauge	Forearm	None	Placebo vs. active magnet
Mayrovitz, 2002 [45]	12	Ceramic magnets	100 mT	Anterior forearm	30 minutes	Laser Doppler	Anterior forearm	None	Compared to pre-exposure and sham on the contralateral arm
Mayrovitz, 2005 [46]	12	Molybdenum magnets	400 mT	Finger anterior	30 minutes	Laser Doppler	Finger dorsum	Decrease	Sham vs. active magnet
Mayrovitz, 2005 [47]	24	Ceramic magnets	100 mT	Finger anterior	20 minutes	Laser Doppler	Finger dorsum	None	Sham vs. active vasoconstrictive responses
Kuipers, 2007 [48]	15	Magnets embedded in a mattress	60 mT	Posterior whole body	60 minutes	Doppler ultrasound	Brachial artery	None	Placebo vs. active
Yan, 2011 [49]	18	Neodymium-iron-boron magnets	223 mT	Finger	30 minutes	Laser Doppler	Finger dorsum	None	Two groups (sham vs. active) with nine per group
Mayrovitz, 2021 [50]	12	Neodymium-iron-boron multi-pole magnets	400 mT	Wrist	45 minutes	Laser Doppler	Finger pulp	None	Sham vs. active magnet

Studies on animals

Table 2 summarizes the major elements of the 13 studies in which an SMF was applied to animals, and some aspects related to blood flow were measured. These studies will be considered in further detail in the discussion section. Regarding the blood flow parameter measurements in these animal studies, six utilized a form of video recording of microscopic images followed by offline tracking of RBC movement that provides a measure of RBC velocity (V_RBC_). If the blood vessel in which these measurements are made does not change, then V_RBC_ changes are proportional to blood flow changes [39,40]. Three of the studies use a method described as microphotoelectric plethysmography (MPPG). This method records the change in opacity of the recorded microscopic field, and it is assumed that an increase in opacity reflects an increase in RBC volume within the microvascular network. As noted in the table, eight studies reported no change or a decrease in response to SMF exposure, while six reported an increase.

**Table 2:** Summary of animal study results Column 1 shows the first author, year of publication, and reference number. SMF is the applied static magnetic field unless specified as the value at the target. V_RBC_ is the red blood cell velocity, SBF is the skin blood flow, and MPPG is the microphotoelectric plethysmography method that measures changes in optical opacity within the microscopically observed microvessels.

First author, year, Ref #	Animal type	Animal studied (N)	SMF source	SMF intensity	Application site	Application duration	Flow measurement method	Flow Measurement site	Mean blood flow change	Comment
Ichioka, 1998 [51]	Rat	20	Superconducting magnetic chamber	8,000 mT	Whole body	20 minutes	Video image processing of venule V_RBC_	Skin fold	Transient increase after exposure	10 exposed to SMF and 10 to shams
Ichioka, 2000 [52]	Rat	50	Superconducting magnetic chamber	8,000 mT	Whole body	20 minutes	Laser Doppler	Skin fold panniculus carnosus	Decrease during exposure	35 exposed to SMF and 15 to shams
Steyn, 2000 [53]	Horse	6	Magnetic wrap	27 mT	Metacarpus	48 hours	Scintigraph images with radio-labeled RBCs for V_RBC_	Metacarpus	None	Sham-treated leg vs. active magnet on another leg
Xu, 2000 [54]	Mice	8	Electromagnet	10 mT	Whole body	10 minutes	Microscopic video image of V_RBC_	Skeletal muscle capillaries	V_RBC_ increase	V_RBC_ increased vs. baseline
Gmitrov, 2002 [55]	Rabbit	8	Neodymium-iron-boron magnet array	250 mT at target	Ear	40 minutes	MPPG	Skin in ear chamber	MPPG signal increase compared to baseline	MPPG signal increased
Li, 2007 [56]	Rat	12	Neodymium-iron-boron magnet	30 mT at target	Trochanter	40 minutes	Laser Doppler	Trochanter skin	Increased	SBF increased vs. baseline
Brix, 2008 [57]	Hamster	8	Neodymium-iron-boron magnet	587 mT at target	Dorsal skin fold	1-60 minutes	Video image analysis of V_RBC_ in capillaries	Striated skin muscle	Decreased	V_RBC _decreased vs. baseline
Strieth, 2008 [58]	Hamster	6	Neodymium-iron-boron magnet	587 mT at target	Dorsal skin fold	1-60 minutes	Video image analysis of V_RBC_ in capillaries	Tumor microvessels	Decreased	V_RBC_ decreased vs. baseline
Strelczyk, 2009 [59]	Hamster	8	Neodymium-iron-boron magnet	586 mT at target	Dorsal skin fold	180 minutes	Video image analysis V_RBC_ in capillaries	Tumor microvessels	Decreased	Decreased vs. a control group
Gmitrov, 2013 [60]	Rabbit	20	Neodymium-iron-boron magnet	250 mT at target	Ear chamber	40 minutes	MPPG	Skin in ear chamber	Increased	Increased vs. pre-exposure
Xu, 2013 [61]	Rat	22	Samarium-cobalt rod	154 mT at target	Fifth caudal vertebra implant	3, 5, and 7 weeks	Near-infrared spectroscopy parameters	Caudal skin and muscle	No change	Active vs. sham-implanted animals
Edner, 2015 [62]	Horse	6	Not specified	90 mT	Back	60 minutes	Photoplethysmography	Back skin and muscles	No change	Active vs. pre-treatment
Li, 2019 [63]	Mice	30	Neodymium-iron-boron magnet array	57 mT, 107 mT, 116 mT at target	Auricle of the ear	60 days	Video images with flying spot tracking for V_RBC_	Muscular-cutaneous tissue	No difference in V_RBC_ between groups	Kept in cages with a magnetic array

Discussion

Human Studies

In 1985, Barker and Cain set out to investigate the claimed vascular effects of a permanent magnet foil [41]. At that time, they used temperature changes in the forearm as a surrogate measure for blood flow changes. In 10 healthy subjects, they wrapped one forearm with a magnetically active foil and the other forearm with magnetically deactivated foil for seven hours while monitoring the temperature every 30 minutes. Their results failed to detect a statistically significant difference in temperature between the arms (p > 0.05). This implies that no differential increase in blood flow could be attributed to the magnetized foil application. Seven years later, a report appeared in the dental literature in which the blood flow effects of magnets used to retain dental prostheses were evaluated [42]. Ten young adult subjects had mini-magnet units placed on one side of their mouths and shams placed on the other side. The magnets were formed from a cobalt-chromium alloy, and mucosal blood flow was measured using the ^133^Xe clearance method. They reported that the control and experimental sides showed no statistical difference (p > 0.05) in blood flow measured up to 45 days after placement.

**Figure 2:**
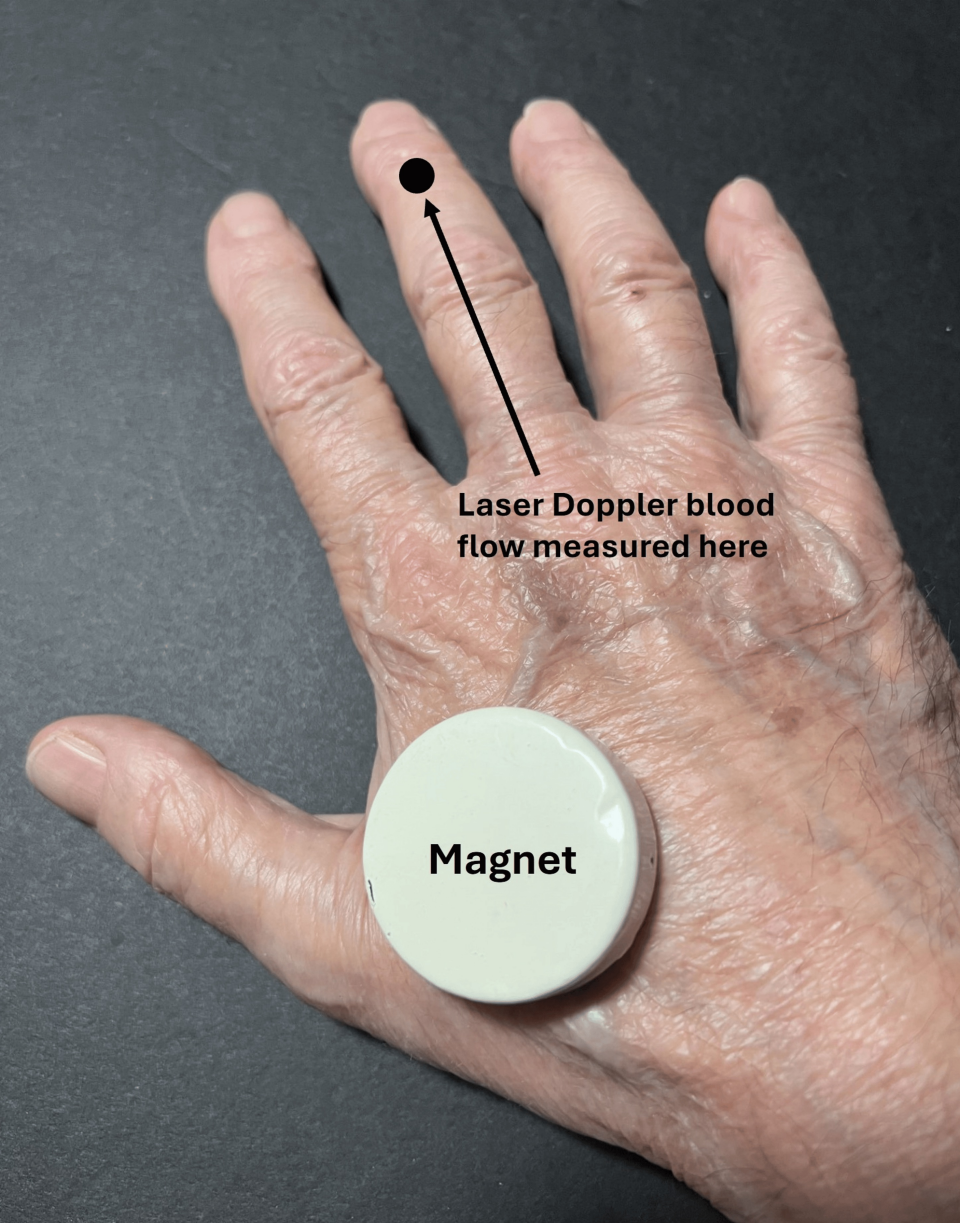
Illustrating the method of reference A ceramic magnet with a surface field of 100 mT was placed on the hand dorsum, and laser Doppler blood flow was measured on the finger dorsum of the third finger. On the other hand, a similar arrangement simultaneously but with a sham magnet. The figure is courtesy of Dr. HN Mayrovitz. [43]

In 2021, laser Doppler methods to assess possible SBF changes due to SMFs were introduced [43]. Finger dorsum SBF was measured when a circular ceramic magnet with a surface field of 100 mT or a sham magnet was placed on the hand dorsum of five subjects, as shown for one hand in Figure 2.

**Figure 3:**
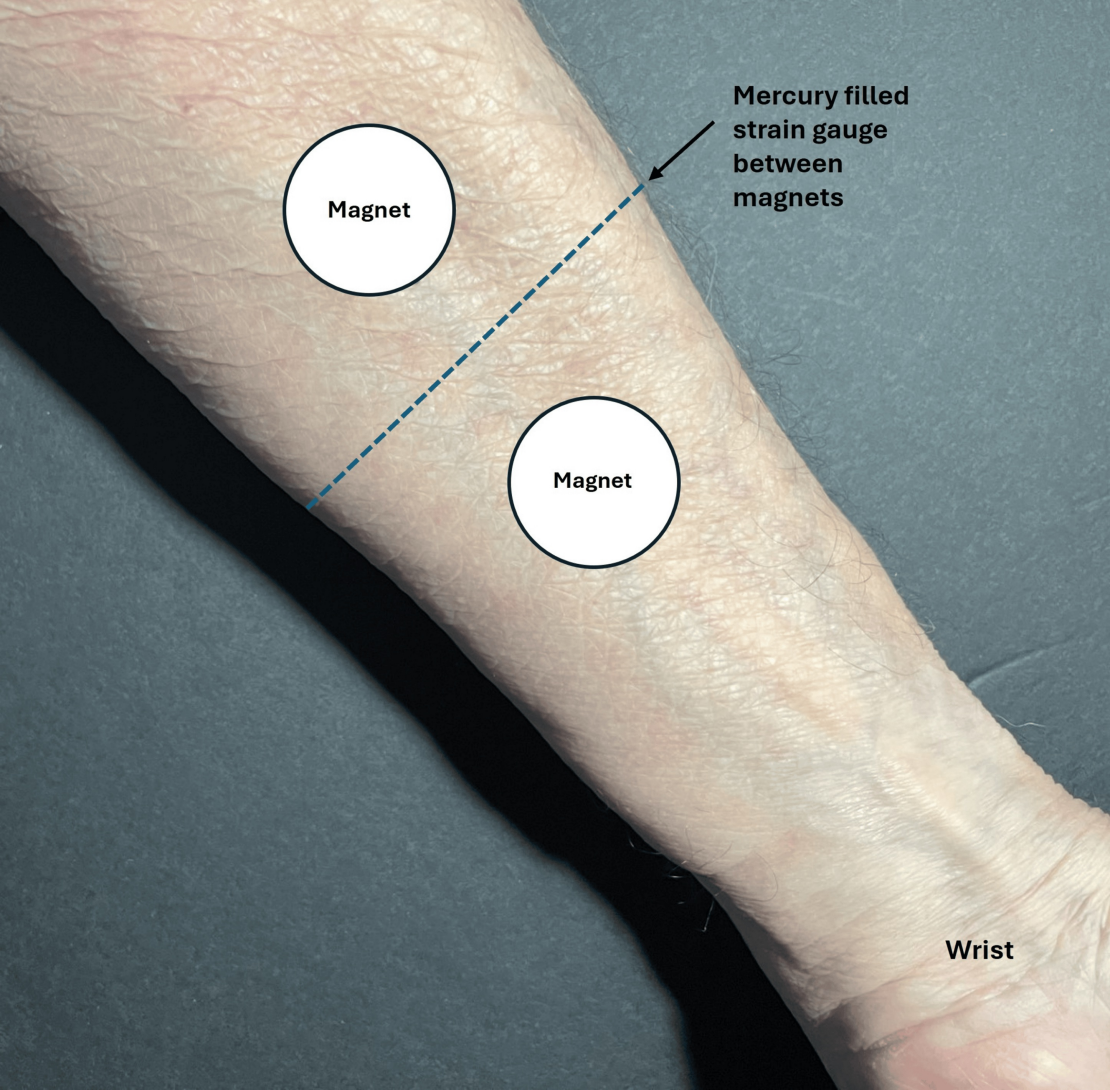
Illustrating the method of reference Two magnets with surface fields of 50 mT were placed on the arm on one day and two sham magnets on another. Blood flow was measured by venous occlusion plethysmography. The strain gauge quantifies changes in limb volume. The figure is courtesy of Dr. HN Mayrovitz. [44]

The active magnet or sham magnet was placed overlying the thenar eminence with the hand resting on the magnet or sham of five additional subjects, with the magnet in contact with the thenar eminence. In addition, on six other subjects, the entire index finger of both hands was scanned with a laser Doppler imager while the hand rested on a magnetic pad with a surface field of 100 mT. The analyses reported by these investigators indicate no difference in SBF response to the active or sham magnet (p > 0.05). Martel and co-workers adopted a different measurement approach [44]. They used venous occlusion plethysmography to measure blood flow in 20 young adult men's non-dominant arms when magnets or shams were placed on the arm at locations similar to that shown in Figure 3.

**Figure 4:**
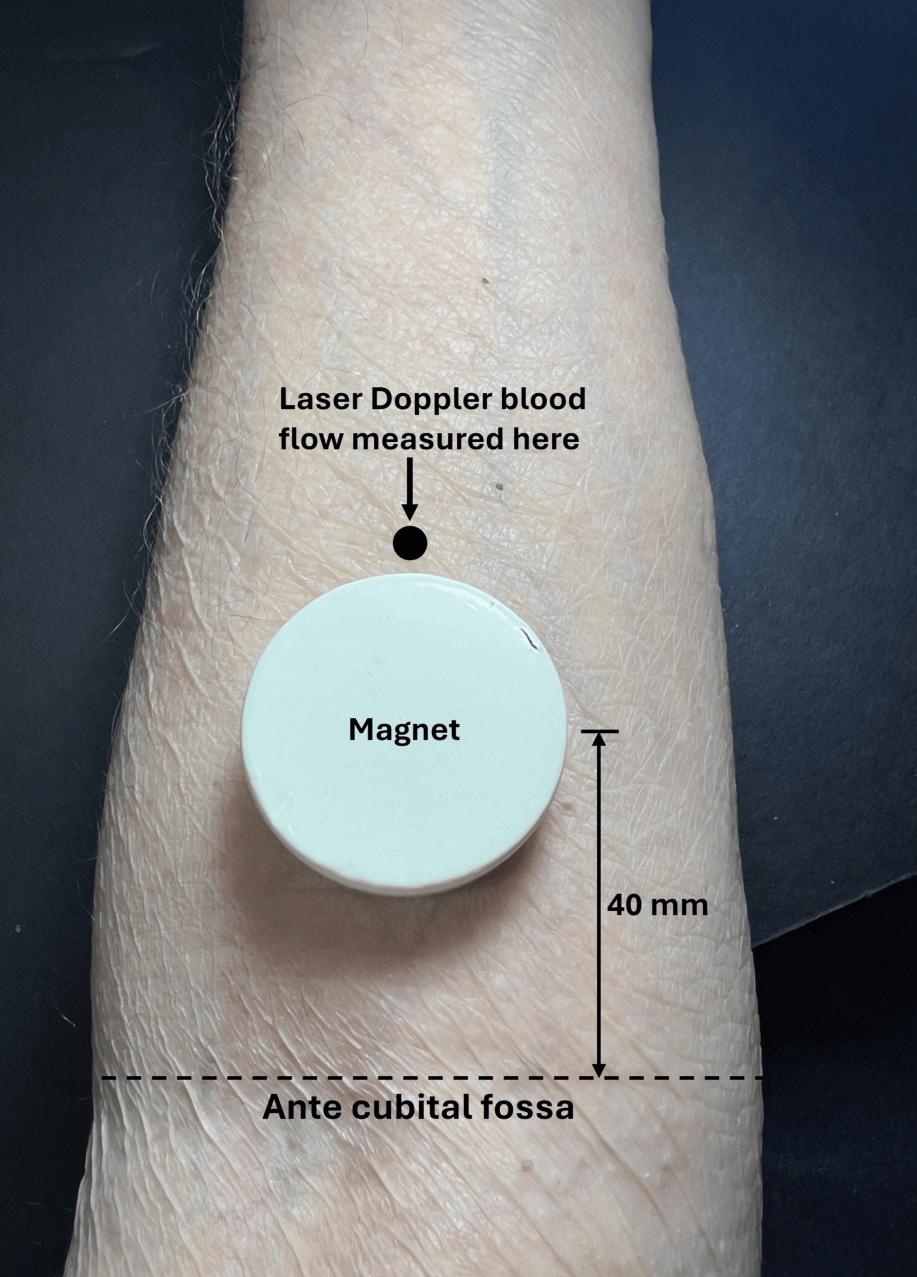
Illustrating the method of reference Laser Doppler blood flow was measured for 30 minutes on both arms simultaneously, with one arm having an active magnet and the other with a sham magnet. The figure is courtesy of Dr. HN Mayrovitz. [45]

The magnets were described as having concentric polarity with a surface field strength of 50 mT and were applied for 30 minutes. They report no significant difference in forearm blood flow between the active magnet and sham treatment sessions. In the same year (2002), the effect of an SMF vs. a sham magnet on forearm SBF was measured in 12 subjects using the configuration illustrated in Figure 4 [45].

**Figure 5:**
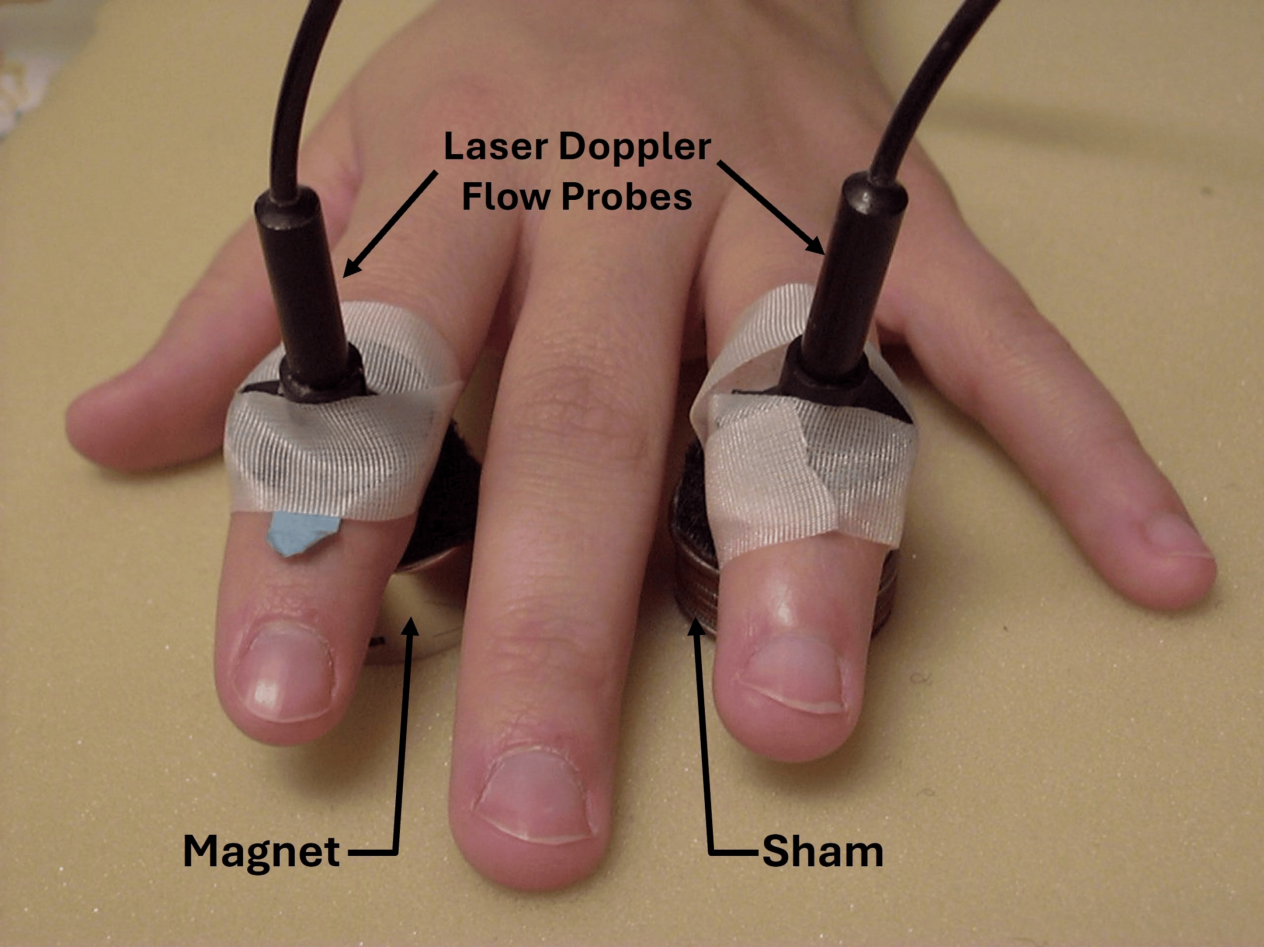
Illustrating the method of reference The index finger was exposed to the magnet for 30 minutes, with the north and south poles facing up for 15 minutes each. The figure is courtesy of Dr. HN Mayrovitz. [46]

The ceramic magnet, with a surface field of 100 mT, was placed on one arm and a sham magnet on the other for 30 minutes while SBF was continuously recorded. The investigators report no significant difference in SBF attributable to the active magnetic field (p > 0.05). Subsequently, possible SMF effects were assessed using a greater field intensity [46]. In this study, a neodymium magnet with a surface field of 400 mT was used in a configuration as illustrated in Figure 5.

Before exposing the index finger to the magnetic field, both fingers rested on shams with SBF recorded for 15 minutes. Thereafter, the index finger rested on the active magnet for 30 minutes, split between the north and south poles facing upward for 15 minutes each. Based on the analysis of the SBF data, these investigators reported that the difference between sham and active magnet SBF indicated a statistically significant decrease in SBF associated with the active magnet (p < 0.05).

**Figure 6:**
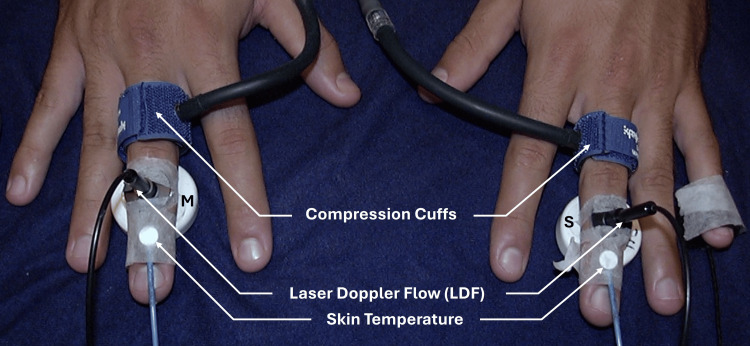
Illustrating the method of reference The middle finger of both hands is initially exposed to shams, and then, one sham is replaced with an active magnet. Changes in SBF induced by multiple inspiratory gasps are compared to evaluate the effect of the active magnet on SBF reduction. The compression cuffs are only used at the end to obtain a biological zero for the SBF calibration. The figure is courtesy of Dr. HN Mayrovitz. [47] SBF: skin blood flow

Because it had been suggested that the vascular effects of an SMF might be dependent on the vasodilatory state [33,34], a study was undertaken to determine the impact of an SMF on SBF changes associated with neurally induced vasoconstriction [47]. The investigators used an arrangement in which the middle finger of each hand was resting on an active magnet or a sham, as illustrated in Figure 6.

In their procedure, both middle fingers were first placed on sham magnets to obtain baseline values for 15 minutes, after which three inspiratory gasps were initiated three minutes apart. These triggered an intense transient vasoconstriction with decreased SBF in both hands. Then, one of the shams was replaced with an active magnet with a surface magnetic field intensity of 100 mT, and the process was repeated after being exposed to the SMF for 20 minutes. After analysis of the responses of 24 subjects, the investigators reported that the magnetic field did not affect the vasoconstrictive response for either SBF or skin temperature (p > 0.05).

**Figure 7:**
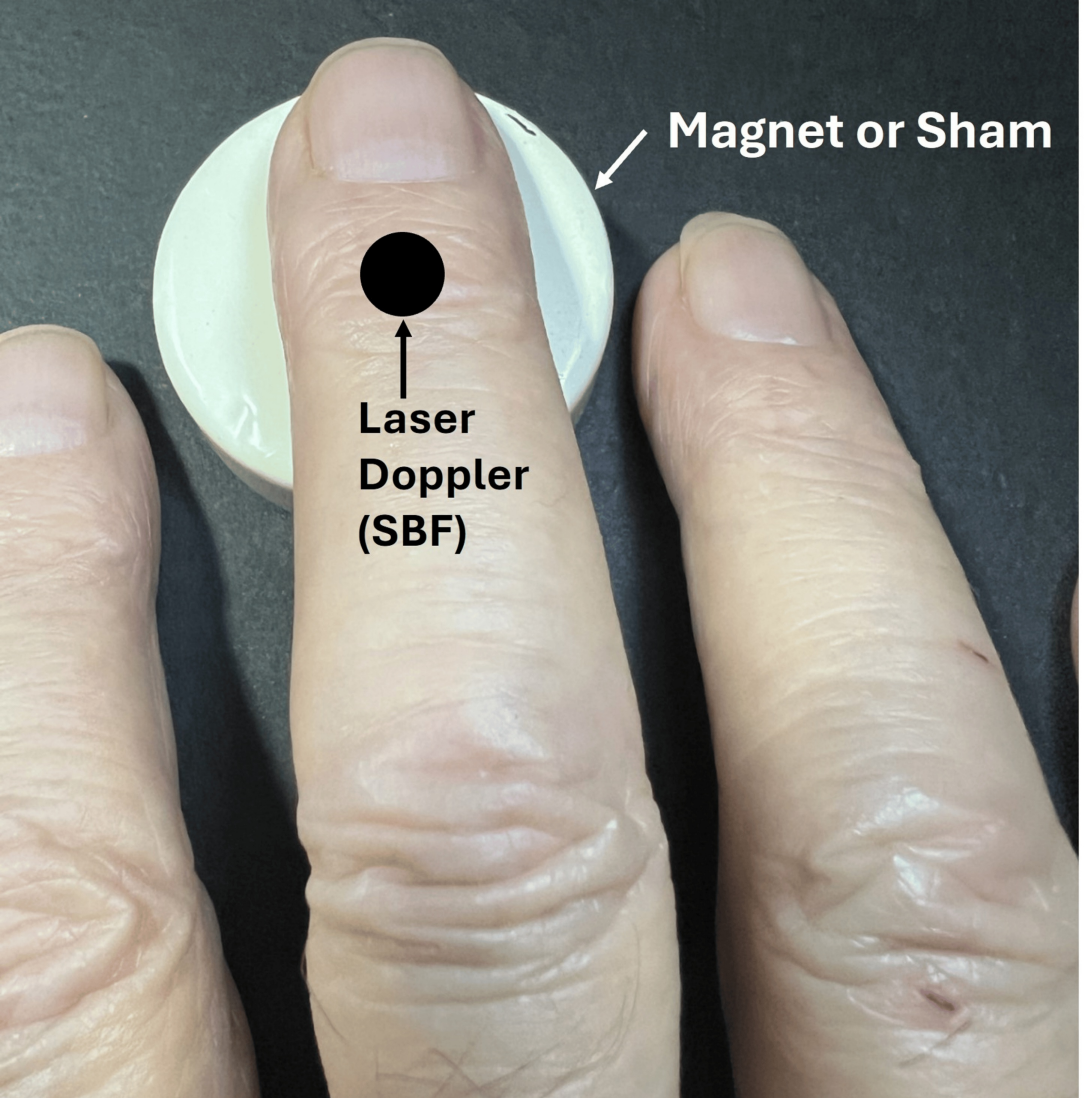
Illustrating the method of reference Nine subjects were exposed to the magnet, which had a surface field intensity of 223 mT for 30 minutes, and nine others were exposed to the sham with SBF recorded continuously for 90 minutes. The figure is courtesy of Dr. HN Mayrovitz. [49] SBF: skin blood flow

A more generalized approach to the question of an SMF effect was taken by Kuipers and co-workers, who investigated the effects of whole-body exposure provided by a mattress into which were embedded 95 magnets, each with a surface field of 60 mT [48]. In this study, several measurement types were made; blood flow was measured in the brachial artery using ultrasound in 15 subjects while lying on a mattress with the embedded magnets and a mattress without the magnets. They report no significant differences in forearm blood velocity after one hour of supine lying on either mattress (p > 0.05). A potential criticism of this study is that it is unclear how far the magnets were positioned within the mattress from the body surface. This is especially relevant since the investigators report that the field intensity was indistinguishable from the Earth's field at a distance of 9 cm from the magnet surface. A more anatomically focused study was conducted by Yan and co-workers, who investigated the impact of an SMF produced by a neodymium-iron-boron magnet on finger dorsum SBF assessed via laser Doppler [49]. Their approach is illustrated in Figure 7.

**Figure 8:**
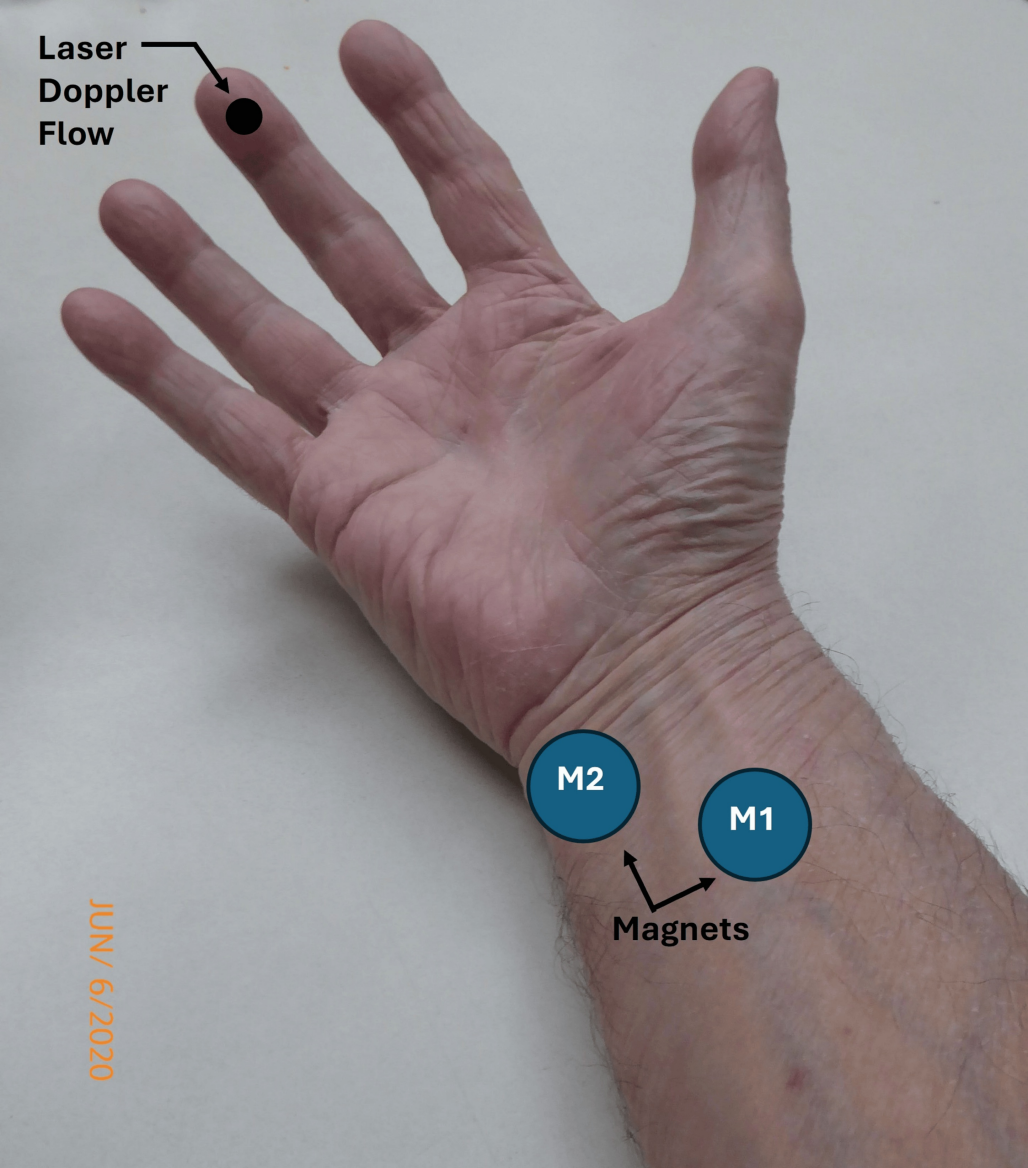
Illustrating the method of reference Magnet M1 was placed over the median nerve and M2 over the ulnar nerve and artery for 30 minutes. Sham magnets were placed at corresponding sites on the contralateral arm. Skin blood flow was measured on the middle finger pulp via laser Doppler. The figure is courtesy of Dr. HN Mayrovitz. [50]

Their protocol consisted of a 30-minute magnet or sham exposure preceded by a 30-minute control and a 30-minute post-exposure measurement. Nine subjects were exposed to the magnet with a surface field intensity of 223 mT, and nine others were exposed to the sham. Based on their measurements, they reported no statistical difference in SBF between the two groups or within each group in each time interval (p > 0.05). The most recent investigation used two concentric neodymium-iron-boron magnets positioned over the median and ulnar nerves with SBF measured via laser Doppler at the middle finger pulp for 30 minutes [50]. In this study, one side had the active magnets positioned, as illustrated in Figure 8, and the other side had two shams positioned at the corresponding anatomical sites.

The maximum perpendicular surface field of the magnets was indicated as about 280 mT, and the tangential maximum was about 200 mT. The investigators reported no statistically significant difference in SBF values between the magnet and sham-exposed sides (p > 0.05).

Commentary on Human Studies

Although none of the 10 human studies reported an increase in blood flow associated with the application of the SMF, four limitations may have impacted the absence of a positive finding: (1) the number of subjects included is relatively small, which affects the study power; (2) the duration of the SMF application of most studies was relatively short, not exceeding about one hour; (3) most studies were done on healthy individuals; and (4) the SMF was delivered perpendicular to the body surface, so the effects of tangential field directions are unknown. There is also a potential issue concerning the magnitude of the magnetic dose. In some studies, the dose at the target was not specified, and it is unknown if a threshold dose is needed for a blood flow effect. Although these provisos may impact the detection of a possible SMF effect, they do not alter the current findings, and no reviewed study has demonstrated a statistically significant increase in blood circulation attributable to an SMF.

Animal studies

**Figure 9:**
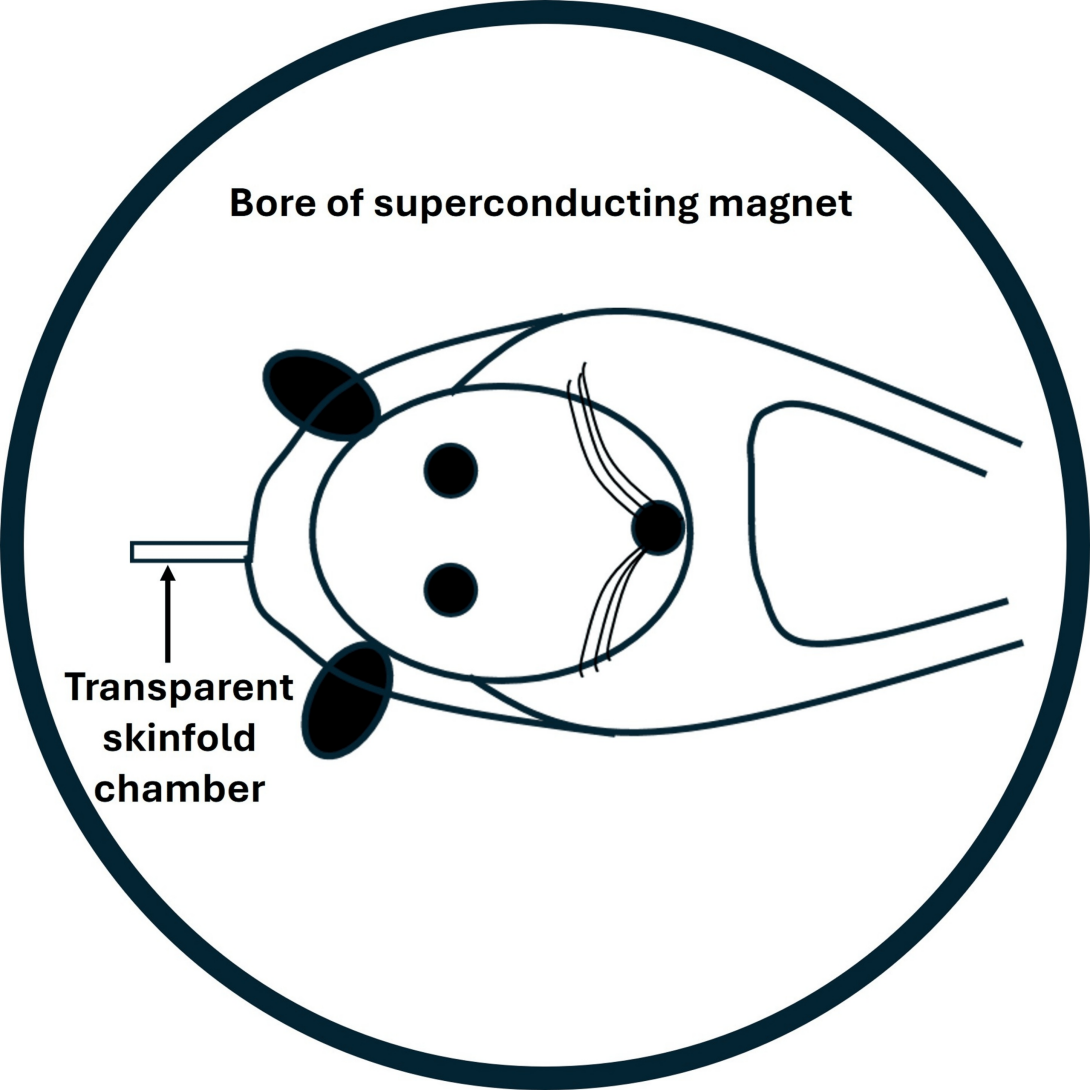
Illustrating the SMF exposure method of reference The rat and transparent skinfold chamber are placed within the bore of a superconducting magnet with a nearly homogeneous SMF of 8,000 mT for 20 minutes. The figure is courtesy of Dr. HN Mayrovitz. [51] SMF: static magnetic field

In 1998, Ichioka and colleagues reported on a study in which the whole body of 20 rats was exposed to a strong SMF of 8,000 mT for 20 minutes [51]. To observe the potential effects of the microcirculation, they used a surgical procedure to create a skinfold transparent chamber. The SMF was created by a superconducting magnet to which the rat with the transparent skin fold was exposed in a cylinder as illustrated in Figure 9.

After pre-exposure measurements of blood velocity in microvessels in the skin fold network for 10 minutes, the rat and the transparent skinfold chamber were placed within the magnet's bore with a nearly homogeneous SMF of 8,000 mT for 20 minutes. Post-exposure measurements were then done. This procedure was completed in 10 rats and in 10 other rats that were handled similarly, except that the magnetic field was not turned on and constituted a sham treatment. Blood velocity increased one to five minutes after exposure in the actively exposed group (p < 0.05). This increase was not observed in the sham-exposed group (p > 0.05). Based on measurements of rectal and skin temperature decreases during exposure, the investigators speculated that the post-exposure transient blood velocity increase was a hyperemic response triggered by a flow reduction during exposure and not directly to the SMF. A study was conducted to follow up on this, using the same exposure method, but measurements were allowed during the SMF exposure using laser Doppler methods [52]. Data from 35 rats exposed to the 8,000 mT SMF for 20 minutes clearly showed a significant decrease in flow during the SMF exposure compared to 15 other rats exposed to sham treatment (p > 0.05). Based on simultaneous skin temperature measurements, the investigators concluded that the decrease in flow may have been caused by a skin temperature decrease associated with the SMF interaction with microclimate within the magnet’s bore.

Measurements from a much larger creature, a horse, showed no significant effect (p > 0.05) of a magnetic commercial blanket on blood flow in the metacarpus of six healthy horses [53]. Magnets within the blanket had a surface field of 27 mT, but at 7 mm from the magnet, the field was no more than the Earth’s field (0.05 mT). Each of the six horses had their legs wrapped with the blanket containing magnets and, at another time, wrapped with a blanket with the magnets removed. Blood flow at each time was measured using radiolabeled RBCs with 75 microcuries of technetium and capturing images of the metacarpal with a gamma counter. Comparisons between the SMF and sham treatment results indicated no significant difference. The investigators considered the low field intensity at the target to not be of clinically meaningful strength.

**Figure 10:**
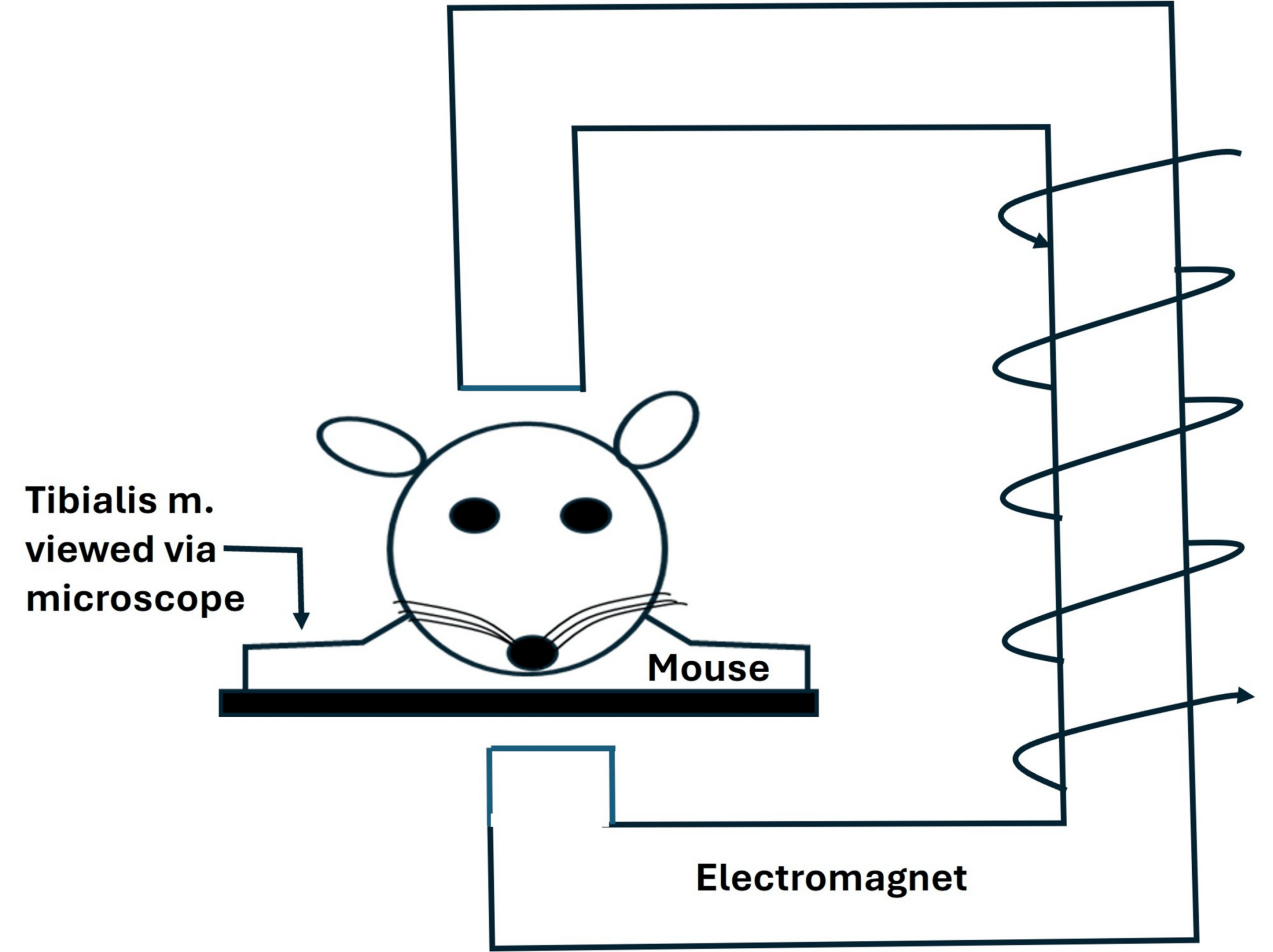
Illustrating the SMF exposure method of reference Red blood cell (RBC) movement in muscle capillaries was recorded and then measured to determine RBC velocity frame-by-frame for 0.3, 1, and 10.0 mT SMF exposures for 10 minutes. The figure is courtesy of Dr. HN Mayrovitz. [54] SMF: static magnetic field

Another whole-body exposure study was done by Xu and colleagues using mice as the experimental animal [54]. Their method is illustrated in Figure 10, in which an electromagnet delivers the SMF to the animal being studied.

RBC velocity in the tibialis anterior muscle capillaries was measured using video recordings of images captured with fluorescence epi-illumination. Three different SMF intensities were used, 0.3, 1.0, and 10 mT, in addition to a sham exposure, each for 10 minutes. The results indicated that the peak blood velocity was increased by exposure to the SMF compared to that measured in the sham treatment (p < 0.05). The largest increase reported was with the 10.0 mT exposure, which resulted in a 45% increase as measured at the end of the 10-minute exposure and then rapidly declined. A potential limitation of this study was that RBC tracking was done frame-by-frame on video playback, limiting the number of capillaries that could be included in the sample. Furthermore, the number of capillaries measured was not indicated. Selecting only the peak velocity rather than the average velocity during exposure may introduce visual bias.

**Figure 11:**
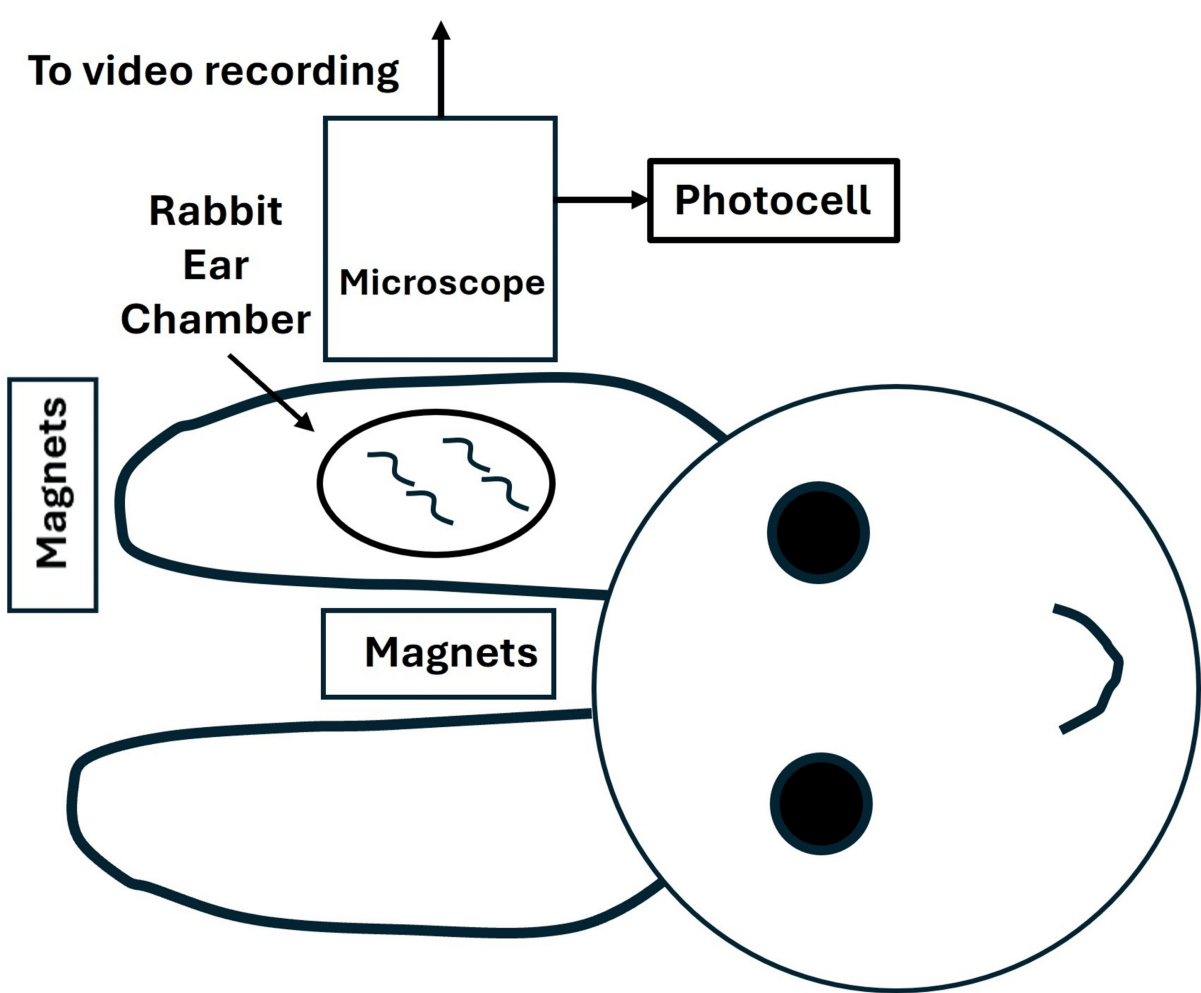
Illustrating the SMF exposure method of reference A group of neodymium magnets produced a 250-mT field strength at the rabbit ear chamber (REC). Changes in the REC's opacity were determined by photoelectric plethysmography. The figure is created by and courtesy of Dr. HN Mayrovitz.  [55] SMF: static magnetic field

A different microvascular observation method employed the rabbit ear chamber (REC) technique. The REC was exposed to an SMF from a group of neodymium magnets that was reported to produce an SMF at the REC of 250 mT [55]. A schematized version of the method used is illustrated in Figure 11.

Changes in the microvascular network within the REC were determined by changes in the opacity of the microvascular field using a photoelectric method. These changes in opacity were used to assess changes in hemodynamics associated with applying the SMF. A typical experiment had a 20-minute control, a 40-minute magnet application, and a 20-minute post-exposure interval. Average values in each 10-minute interval were compared to the first 10-minute interval for experiments using and not using the magnets. Based on data from eight animals, the results indicated a decrease in the opacity signal with time if no magnet was used (p < 0.05) but an increase in opacity (p < 0.05) when the SMF was applied. This resulted in a statistically significant difference (p < 0.05). Although the authors state that this represented an increase in blood flow, it should be noted that the parameter they measured was not blood flow directly.

A novel use of laser Doppler measurements of SBF to test the effects of an SMF targeted the trochanter region of mice that had experienced loading on their trochanter for five days at a pressure of about 100 mmHg [56]. Although much of this study was concerned with spectral analysis of flow motion, the investigators reported that the average SBF measured during 40 minutes of exposure to an SMF of 30 mT at the target resulted in an increase in SBF only in those animals that had been subjected to the effects of tissue loading (p < 0.05). In the animals that had experienced the five-day loading, the increase in SBF was 19% compared to pre-exposure (p0.05) but was not sustained beyond the exposure interval. No effect on SBF was observed when the SMF was applied to animals that had not previously experienced the effects of tissue loading (p > 0.05). These findings suggest that some changes attributable to the loading altered the responsiveness of the underlying vasculature to the SMF.

In contrast to an increase in SBF, an SMF of 587 mT applied to a hamster dorsal skin fold preparation resulted in a reduction of 41% (p > 0.05) [57]. The preparation method used was similar to that shown in Figure 9, except instead of a rat, a hamster was used. Using fluorescence and a continuous video recording, the measured quantity was the RBC velocity in skin muscle capillaries. The reported data is based on a reasonable sample that included eight regions of interest within the recorded microvascular image and three vessels within each region. An important aspect of this study was that the animals were not anesthetized, so the SBF decrement was observed in the awake state, and potential impacts of anesthesia were absent. Another notable finding of this study was that the SMF's impact was rapid and reversible. A decrease in RBC velocity (p < 0.05) was also reported when implanted tumors were exposed to an SMF of 587 mT [58]. The tumors were implanted in the hamster dorsal skin fold preparation, and RBC velocity was measured using fluorescence microscopy. RBC velocity was reported to be reduced by 40% (p < 0.05) soon after application of the SMF and was also reversible. A similar finding using a similar method was subsequently reported for implanted tumors [59].

In 2013, an additional series of experiments using the REC method were done [60], as illustrated in Figure 11. Animals were exposed for 40 minutes to a magnet array that caused an SMF of 250 mT at the target. In another group, shams were used for the same amount of time. Using changes in the MPPG as an indicator, they report a 17.8% increase during the SMF exposure and a decrease during sham exposure (p < 0.05). There are two potential issues associated with this report. The first is that the parameter MPPG is not a proven indicator of blood flow. The second relates to the statistics used by the investigator. A paired t-test was used based on the number of experimental runs, indicated as 20, rather than the number of animals used.

A longer-term application of an SMF was attempted when magnetized and unmagnetized rods of samarium-cobalt were implanted in the caudal vertebrae of rats for up to six weeks, and caudal artery blood flow parameters were measured with near-infrared spectroscopy (NIRS) [61]. The maximum surface field strength of the magnetized rod was 160 mT. The investigators reported no significant differences between magnetically and sham-implanted caudal artery blood flow parameters measured three, five, and seven weeks post the initial implants (p > 0.05). It should be noted that blood flow was not measured in this study. Instead, the NIRS method provides parameters related to oxygen utilization within the caudal muscle. Thus, the absence of a change in these oxygen-related parameters suggests but cannot directly demonstrate that there was no change in actual blood flow.

In 2015, another group aimed to investigate the impact of a magnetic blanket on muscular blood flow in horses [62]. Data were obtained on six horses exposed to a magnet-embedded blanket at one session and a blanket without magnets applied at another session. The application lasted 60 minutes. An index of blood flow was obtained using a photoplethysmographic (PPG) sensor operating together with near-infrared wavelength light-emitting diodes. This measurement assumes that the peak-to-peak change in the PPG signal measures pulsatile blood volume and flow. The investigators report no statistical difference (p > 0.05) in either group during the treatment interval.

The impact of long-term SMF exposure was evaluated in mice housed in a cage exposed to peak intensities of approximately 58, 107, and 116 mT for 60 days [63]. In this study, 10 mice per group were housed together at each SMF intensity, and subsequently, each was evaluated for blood velocity using video imaging and RBC tracking. Results showed no difference in auricular muscular cutaneous blood flow compared to those similarly housed without an SMF (p > 0.05).

## Conclusions

Concerning human studies, none showed an increase in blood flow (p > 0.05), and one showed a decrease in flow (p < 0.05). One of the animal studies showed a transient post-exposure increase (p < 0.05) that was later explained due to an actual reduction during SMF exposure. Four studies showed a decrease (p < 0.05), four showed no change or difference from sham-exposed animals (p > 0.05), and four reported an increase (p < 0.05). It is concluded that claims of an SMF providing an increase in blood flow are not supported by human studies and not well supported by animal studies. However, this does not close the door to a possible effect for at least four considerations that may have impacted the absence of a positive finding in human and animal studies: (1) the number of subjects or animals included is relatively small, which affects the study power; (2) the duration of the SMF application of most studies was relatively short; (3) most studies were done on healthy individuals or animals; and (4) the SMF was delivered perpendicular to the body surface, so the effects of tangential field directions are unknown. There is also a potential issue concerning the magnitude of the magnetic dose since it is unclear if a threshold dose is needed for a blood flow effect. Although these provisos may impact the detection of a possible SMF effect, they do not alter the current findings, as no reviewed human study and few animal studies have demonstrated a statistically significant increase in blood circulation attributable to an SMF. Thus, the experimental evidence does not support the clinical use of an SMF to improve blood circulation.
